# solidarity through mail-based participatory visual research: exploring queer and feminist futures through an art, activism and archiving project with 2SLGBTQ+ youth amidst COVID-19

**DOI:** 10.1177/01417789231205297

**Published:** 2023-12-11

**Authors:** Casey Burkholder, Katie MacEntee, Amelia Thorpe

**Keywords:** 2SLGBTQ+, Atlantic Canada, solidarity, participatory visual research, feminist futures, zines, dioramas

## Abstract

The COVID-19 pandemic posed a logistical problem to our normal ways of engaging in participatory visual research. Our in-person art, activism and archiving with 2SLGBTQ+ Atlantic Canadian youth pivoted to use distanced engagement strategies that met the demands of the pandemic. We sought to create networks of solidarity while we were apart. Monthly, over the course of a year, we mailed out themed packages of art supplies and directions to fifty-five 2SLGBTQ+ youth situated in the Canadian provinces of New Brunswick, Nova Scotia, Prince Edward Island and Newfoundland and Labrador. Participants then created the artworks, photographed them and contextualised them through text. While the resulting co-curated digital archive includes multiple mediums, here we focus on the participants’ zines and dioramas for what they taught us about 2SLGBTQ+ youth’s identities, activism, beliefs, friends, home, family, fears, strengths and futures. The digital archive of our artwork deconstructs, explores and affirms identities and functions to build solidarity during a time of increased isolation. We argue that collaboratively building the digital archive was a feminist act of reclamation and a declaration of youth queer activism.

## Introduction

COVID-19 encouraged us to engage in solidarity through research for social change in new ways. Originally conceived as an in-person art, activism and archiving project, we pivoted our participatory visual research project, Pride/Swell, with 2SLGBTQ+^1^ Atlantic Canadian youth to meet the demands of the pandemic. We sought to create networks of solidarity while we were apart. Monthly, over the course of a year, we mailed out themed packages of art supplies and directions to fifty-five 2SLGBTQ+ youth situated in the Atlantic Canadian provinces of New Brunswick, Nova Scotia, Prince Edward Island and Newfoundland and Labrador. While we created twelve art practices in the year, in this article we focus explicitly on two: zines and dioramas. We made our zines about our intersectional identities and relationships to power. Our dioramas imagined queer environmental futures. Our resulting co-curated archive of these zines and dioramas (see Burkholder, Hamill and Thorpe, 2021) teaches us about 2SLGBTQ+ youth’s identities, activism, beliefs, friends, home, family, fears, strengths and futures. The co-curated digital archive of our artwork deconstructs, explores and affirms identities and functions to build solidarity during a time of increased isolation. Collaboratively building the digital archive through enthusiastic and explicit consent practices was a feminist act of reclamation and a declaration of youth queer activism.

Our inquiry in this article centres on two questions: What did 2SLGBTQ+ solidarity and feminist futures-making look like in our project? What did we notice about the ways that solidarity manifested through distanced art, activism and archiving with 2SLGBTQ+ youth? These questions ground our discussion of Pride/Swell within the first iteration of the COVID-19 pandemic (2020–2021), which was particularly isolating for 2SLGBTQ+ Atlantic Canadian youth living under lockdowns and in rural and remote communities. We highlight how the production, sharing and archiving of 2SLGBTQ+ youth art adds to methodological discussions of exhibiting and digital archiving as a form of activist intervention and solidarity-building within a contemporary moment marked by separation and lacking connections within 2SLGBTQ+ communities. Our inquiry also centres experiences of failure. Disengagement, uneven participation and awkwardness also permeated our attempts to forge queer solidarities and imagine queer and feminist futures. Engaging in participatory visual research through the mail is an original contribution to scholarly understandings of participation in virtual and distance-based projects.

## Context

Pride/Swell was an art, activism and archiving project (2020–2021) that sought to create communities across provincial borders between queer, trans and nonbinary youth aged 15 to 25 years old located in the Atlantic Canadian provinces of New Brunswick, Nova Scotia, Prince Edward Island and Newfoundland and Labrador. We sought to create communities of 2SLGBTQ+ solidarities through purposeful activities that sought to highlight commonalities between participants during this time and within young people’s home spaces. However, as Anne-Marie Fortier writes of queer diasporas, building on Sara Ahmed’s (2000) work:

the enduring appeal of community as ‘being in common’ needs to be scrutinized as well. More specifically, how are communities brought together? What are the social processes involved in imagining and constructing a queer diasporic community? How does diasporizing the queer produce a ‘community’ that involves the movement of some bodies though the fixing of others (Ahmed, 2000)? (Fortier, 2002, p. 7)

Fortier’s problematising of queer community is especially relevant in our attempts to build community across rural and urban geographies, and across generations. We take on Fortier’s questions as we explore how we have built solidarities between the youth as well as between the youth and the research team. While our participants were differently located across class and ability^2^ and age (15 to 25 years old), the majority of participants were White.^3^ Our research team—including the three of us and our collaborators Brody and April—are all White, and each identify differently: trans*, cis and queer. The problem of Whiteness and homonormativity in queer spaces is not new—Rae Rosenberg (2017, p. 139), drawing on Michael Brown (2013), argues that ‘queer geographers and geographers of sexuality have characterized gay urban placemaking as “a zero-sum game of identity and territoriality [wherein the] gains of some marginalized identities come at the exclusion, domination, or oppression of some other” (Brown, 2013, 460)’. While our work is rural and Atlantic Canadian, and despite our efforts to build community and solidarity, we are cognisant of the ways in which our project failed to engage many racialised 2SLGBTQ+ youth throughout our project. We suspect the Whiteness of many of our participants and research team limited our ability to appropriately and effectively engage a more diverse group of youth and may have excluded some racialised and Indigenous youths’ engagement.^4^ Indigenous, Black and racialised 2SLGBTQ+ persons have long faced marginalisation within and beyond 2SLGBTQ+ communities and spaces through pervasive discourses of homonormativity that reinforce compulsory Whiteness. The problem of Whiteness in queer and art-activist spaces in Atlantic Canada is a limitation of any claims we may make about solidarity-building in this project (Ahmed, 2007; Burkholder *et al.*, 2021).

## Theorising queer solidarities

We work with Jonah Winn-Lenetsky’s (2015, p. 4) definition of solidarity as they write, ‘solidarity [is] a mutual investment in the struggles of another identity group that is premised on identification and the desire for reciprocity. Solidarity can take on the form of real world mutual understanding and reciprocity, or can be imagined and performed’. Winn-Lenetsky (*ibid.*, p. 22) continues to suggest that ‘solidarity [is a useful framing] … to work toward mutual investment and understanding in one another’s struggles’. We are also profoundly grateful for the theoretical contributions of José Esteban Muñoz (2009, p. 1) whose work presents us with the compelling idea that ‘we must strive, in the face of the here and now’s totalizing rendering of reality, to think and feel a then and there’. Muñoz (*ibid.*, p. 1) continues, ‘we must dream and enact new and better pleasures, other ways of being in the world, and ultimately new worlds’. Queer folks do this work of queer futurity—of reimagining and questioning and dreaming forward new worlds in their everyday. Pride/Swell constantly does this, too. We ‘dream and enact new and better pleasures, other ways of being in the world, and ultimately new worlds’ (*ibid.*). Imagining queer and feminist futures—those that centre joy and care—through a theoretical and methodological orientation towards queer joy offers us ways of imagining these new and better pleasures, through art-making and queering the world through questioning the present and the past.

This work is joyful, do-it-yourself (DIY), contextual and made clearer through the solidarities we have built throughout the project. Queer and trans joy ‘flies in the face of cis heteronormativity. It says, “we will not buy into these toxic power dynamics. We will not live our lives according to these societal confines around gender roles and sexual norms. But instead, we will live in ways that are about connectedness, humanity, pleasure, and reciprocity”’ (Wright, 2023). In their exploration of queer trans femmes and nonbinary people in Australia’s punk scenes, Megan Sharp and Pam Nilan (2017, p. 71) found that solidarity-building looked like a subversion of patriarchal expectations, where folks ‘mobilised community-building through the politics of Do-It-Together (DIT) as a radical reshaping of the traditional punk ethos of DIY (Do-It-Yourself)’. Our solidarity-building borrows from DIY traditions as we sought to shift our methods with 2SLGBTQ+ youth during a time of increased social isolation through mutual engagement, creation and personal investment in the collective project. Our engagement with the same monthly art prompts invited us to share our lives, identities and politics through individual creation and collective discussion (monthly Zoom meetings).

Our vision of queer solidarities includes deep theorising about care. We centred feminist care and queer joy in our project through: our commitment to ongoing informed consent from all participants; our efforts to meet youth where they are; and our creative theorisations of care through numerous monthly prompts that centred identity, community, ecology and more. As a mail-based art project, we engaged with our participants within the intimate space of their homes. We actively built solidarity with youth inside this private space through both our monthly packages (packaged according to each participant’s needs) and our online meetings. Our creative prompts took up questions of what self-care looks like, what queer environmental futures may hold and what community means. We were inspired by what our colleague Dr Sabine Lebel has queried:

How can we resist the neoliberal quest to download all care to the individual … How can self-care be connected to care for self, community, and environment? Thinking a lot about care practiced by queer communities. Thinking about what queer ecologies can tell us about knowing our webs of oxygen, food, water, community and chosen family.^5^

Our engagement in solidarity is a way to explore queer communities through art production and the creation of digital archives.

## Methods

Participatory visual methods offer opportunities for high levels of impact, participant engagement and social transformation *as part of the research process* (Mitchell, De Lange and Moletsane, 2017; Burkholder, Hamill and Thorpe, 2021; Kendrick, MacEntee and Flicker, 2021). Visual methods can make policy engagement more accessible—through a provocative photograph or one-minute video—by sharing media outputs amidst multiple communities. Pride/Swell was also a project where we sought to engage in a multidisciplinary inquiry through an intersectional lens anchored in queering participatory visual approaches through the mail with 2SLGBTQ+ youth (Fenge, Jones and Read, 2010; Burkholder, Hamill and Thorpe, 2021). 2SLGBTQ+ young people were involved as producers as well as co-disseminators of the work through the development of a digital archive, https://prideswell.org, and the sharing of art pieces on social media (see also Burkholder and Thorpe, 2022), community screenings, exhibitions and public discussions. We see these practices as opportunities to bring a DIY ethos into methods of art production, exhibition and archiving in research for social change framework.

Drawing from the punk scene, DIY methods increase accessibility for artistic creation, queer traditional arts practices and create space for individuals to respond to dominant cultures that marginalise 2SLGBTQ+ identities. In other words, DIY as a material practice is inherently queer. Sharp and Nilan state, citing Ahmed (2004), that:

we propose that the negative *affect* of discomfort drives the positive *affect* of solidarity. Ahmed (2004) identifies the pervasiveness of discomfort as queer feeling or affect, because queer people are compelled to embrace norms that do not fit them and hence live parts of their lives by narratives whose discourses they neither endorse nor embody. (Sharp and Nilan, 2017, p. 72)

Pride/Swell sought to make art in response to structural injustice and inequality, which contributes to transforming and disrupting conventional thinking about youth participation, activism and gender and sexuality, in the present and future.

Syrus Marcus Ware posits that:

centering QTBIPOC (Queer, Trans, Black, Indigenous, Person of Colour) narratives, there is a new entry point and an interruption to the Whitewashing of queer and trans historical accounts. This shift lends the ability to ‘[re]consider the past, presents, and futures’ of racialized queer and trans communities, guiding prospective activism and offering meaningful ways of imagining collective futures. […] Who gets to tell histories shapes the futurity of marginalized communities, and especially racialized queer and trans narratives. (Ware, 2017, p. 172)

Furthermore, we recognise the theorising of Atalia Israeli-Nevo (2017, p. 45) who, drawing on Muñoz (2006), critiques the notion of queer futures because of the enduring problem of Whiteness: ‘the idea of a queer future, anti-future and even time itself is embedded within a white middle-class imperative in which white queers have a future in which they can live safely and (relatively) unharmed, and it is forgotten that queers of colour do not have this kind of imaginable temporal horizon?’. What kinds of claims about solidarity can we make when our work continues to centre White queers? What kinds of claims about solidarity can our team make in a queer research space when our work continues to centre White queers? We take these ideas together as we explore solidarities forged via the mail and DIY with 2SLGBTQ+ youth who were, alongside the research team, majority White.

Our work at a distance centred the needs of queer, trans and disabled participants, but did not engage deeply with politics of antiracism. As critiqued in an article that sought to take the antiracist posturing of White academics from intellectualising to action:

Are we making and supporting decisions in our professional practices that de-centre privilege by inviting perspectives and ceding public spaces to peoples, views, experiences, and actions that can best support an ethics and politics of anti-racism? Are we thinking about what it might mean to do this work in unceded and unsurrendered territories, on the stolen lands of Indigenous peoples? Are we creating solidarities with others in strategizing to do the same? Can our scholarship claim to accomplish much beyond elevating internal, self-involved dramas of anti-racism for our own edification? (Saul and Burkholder, 2020, p. 15)

We do not think our claims of solidarity-building can claim a politics of antiracism at this stage of the Pride/Swell project, though in later iterations (2022–2023) we more concretely have worked to create antiracist art-making spaces. We have worked to trouble our praxis, have continued engaging in land acknowledgements that underscore our commitment to action, and have actively sought out and paid Black, Indigenous and radicalised artists to share their expertise and knowledge with our group. We further continue to create more inclusive and diverse spaces for participants and research team members who are racialised. This is an ongoing process. We continue to question and explore the ways in which we facilitated the mail-based participatory art-making component of Pride/Swell to make it more responsive to the lived experiences of 2SLGBTQ+ youth in the Atlantic provinces.

### Confidentiality and ethics

Our study departs from current participatory research practices, which most often happen in face-to-face contexts (Mitchell, 2011; Mitchell, de Lange and Moletsane, 2017). While mail-based participatory visual research offers original contributions to the study of participation in distance-based projects, it also raises new concerns regarding ethics and confidentiality. The practice also includes particular ethical considerations around authorship and ongoing and enthusiastic consent (Lenette *et al.*, 2018). Consent was paramount to our project, and all participants maintain full control over their artistic work, including which pieces were shared digitally with the research team and with broader publics. Participants decided if they wanted to share their works under a pseudonym or use their real names. Participants retain the right to remove their art from the project and the collective archive at any time. Following an explicit model of checking-in based on participant consensus outlines, we follow up with participants annually about their arts inclusion in the archive. These participant-centred strategies make visible ways that DIY art-activism and solidarity-building through archiving can be made more accessible for and with communities who are socially and geographically marginalised or otherwise excluded from archival processes (Ware, 2017).

In what follows, we explore two of the art practices that our participants engaged in: zine production in response to the prompt ‘I am ____ and ____ and ___’, and dioramas that responded to the prompt ‘imagining queer environmental futures’. We highlight these practices to show how we sought to forge solidarities by examining the present (our identities) and also our imagined queer and feminist futures. We then describe the co-curation of digital archives and the ways in which we engaged in space-making and solidarity-building through virtual meetings.

### Zines

Zines are an intrinsically feminist practice (Piepmeier, 2009; Lymn, 2013; Creasap, 2014; Goulding, 2015). The DIY print productions share stories, drawings, images, collage and things that matter to the producers that make them. Harry Hoke (2019, p. 16) suggests that ‘zines sit at the intersection of artmaking, creative writing, political pamphlet, and community message board to freeze the ideology of a community at a single moment … Zines are communal; zines are textual, visual, temporal, and spatial; zines have unfettered opinions and lots of typos’. Zine scholar Mimi Thi Nguyen (2012) argues that zine production must be explored intersectionally, highlighting issues of marginalisation beyond a single social category. Drawing on Nguyen in her chapter on facilitating research for social change through zines shared via Instagram with Asian migrant women in New Zealand amidst the COVID-19 pandemic, Helen Yeung (2022, p. 100) argues, ‘Migrating my feminist self-publishing framework to Instagram felt intuitive … I wanted to ensure the preservation of acts of subversion, self-representation, and storytelling as found in DIY publishing, and relay these approaches into a digital space’. We work with zines as a queer archival practice (Boatwright, 2019)—a way of storing and sharing information in and across communities.

**Figure 1:**
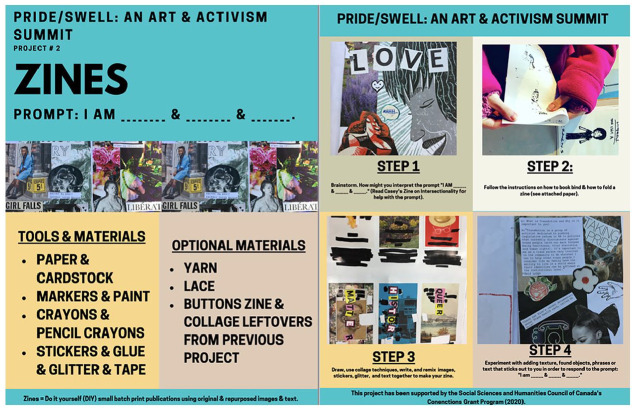
Zine instructions *Source*: Casey Burkholder, 2020, Fredericton: Pride/Swell

To engage the Pride/Swell makers, Casey created packages of art supplies—paper, cardstock, markers, paint, crayons, stickers, glue, glitter and tape (see Figure 1)—that were sent to each of the fifty-five participants’ homes, as well as to the research team. She also shared a short video about how to make a zine, which highlighted how she took up the prompt (see also Burkholder, Hamill and Thorpe, 2021).

### Dioramas

**Figure 2:**
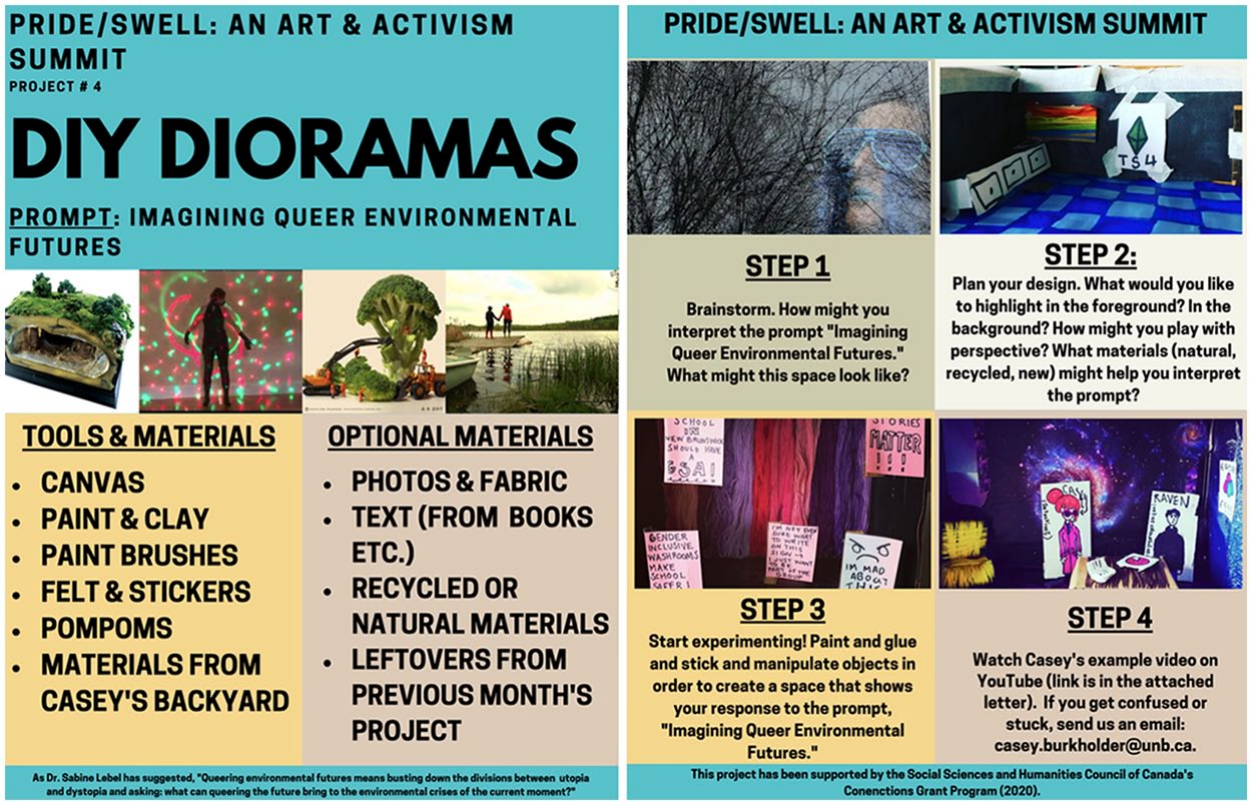
Diorama instructions *Source*: Casey Burkholder, 2020, Fredericton: Pride/Swell

Dioramas are three-dimensional representations of space and have been used within the museum sciences and in social studies education (Gray, Rule and Gordon, 2019). They have not been used in participatory visual research methods, though some of the techniques used within diorama construction (e.g., collage) have been used extensively within research that engages in participatory visual inquiry (Hamill, 2020). In our project, participants received a package that included mixed materials: canvases, leaves from Casey’s backyard, paint brushes, paint, pompoms, felt, stickers and clay (see Figure 2). Although each participant received similar materials, the pieces that emerged from the processes took up different notions of environmental queer futurities, and each of them looked deeply different.

### Co-curated digital archiving

**Figure 3:**
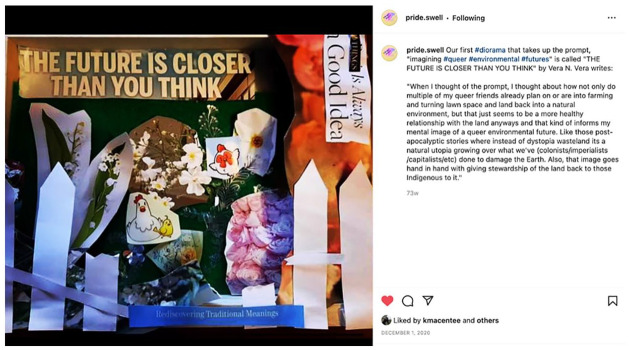
*The Future Is Closer Than You Think*, by Vera *Source*: Vera, 2020, Fredericton: Pride/Swell

Engaging in solidarities in virtual spaces included providing multiple avenues for collaboration and connection. Pride/Swell makers created art in their homes and opted to share—or not share—the pieces that they created by photographing or scanning their works and emailing these images to Casey. Participants would share how and where they wanted their pieces archived digitally. Casey would check in with participants to find out if they wanted their artworks shared on the Pride/Swell website, Facebook, Twitter and/or Instagram accounts.^6^ Participants opted into each space, and some participants titled their works and offered an interpretation of the prompt through text. Some participants chose to archive their work on Instagram and Facebook as well as our project website, while others opted to archive only on our dedicated webpage or only for our internal research team to view (see also Burkholder, Hamill and Thorpe, 2021). Some participants chose to share some while others chose not to share any of their artworks. The Pride/Swell website itself became a virtual archival space to build solidarity within and beyond the project by sharing and affirming 2SLGBTQ+ youth’s experiences and identities through art. We created the digital archive based on participant feedback in our monthly Zoom meetings, opting to structure the website by art practice. We saw solidarities emerge in online spaces as participants and larger publics had the opportunity to respond to and comment on the art pieces (see Figure 3). The public nature of this digital archive encourages empathy and engagement on the part of the viewer, while promoting increased understanding of the struggles and joys of 2SLGBTQ+ youth involved (see Burkholder, Hamill and Thorpe, 2021).

### Virtual meetings and analysis

As a part of our engagement with participants, we hosted monthly Zoom meet-ups where participants would talk about their thoughts on the art shared, and the research team and guest artists would describe the upcoming art practice. This is where our participatory analysis began to take shape. We began each Zoom meet-up with a land acknowledgement and introductions, and arranged for older queer artists who worked in the medium we were exploring to attend the Zoom calls and speak about their work.^7^ When we made dioramas, we hosted Dr Sabine LeBel (2022) whose artistic and scholarly work engages the notion of queer environmental futures—an imagined space somewhere between utopic and dystopic conditions where queer communities thrive as they have always done.^8^ Engaging artists in this way was a form of solidarity-building; we as a research team sought to highlight queer adult artists thriving in our communities as a way of creating solidarities and showing examples of queer futurities. We used the Zoom space to strategise, analyse and choose prompts and art practices for upcoming months. At the same time, these Zoom meetings had lower attendance—although we had fifty-five participants throughout the year making art and receiving packages, we never had more than fifteen participants attend a Zoom meet-up, and it was more often two or three participants alongside the research team and the guest artists. We noticed that some participants were more comfortable listening or participating with their cameras off. Some participants engaged in dialogue exclusively through the chat function, while others kept their cameras on and actively discussed their thoughts. We recorded each Zoom meeting and emailed all participants an audio recording, so that they could engage or not engage with what was shared in these spaces. All of these modes of engagement—including not attending and not listening—are recognised as forms of participation (Switzer *et al.*, 2021).

## Networks of solidarity that emerged

Through the virtual meetings and through our co-curation of the website and social media pages as explicitly queer archival spaces, we articulated our queer and feminist futures. In preparation for the writing of this article, we—Casey, Katie and Amelia—looked back at the transcripts of our Zoom conversations, as well as the Instagram posts that we made with participants, in order to explore the notion of solidarity and how it emerged in the project. We classified these networks into four categories based on people and place.

### Networks between urban and rural youth

**Figure 4:**
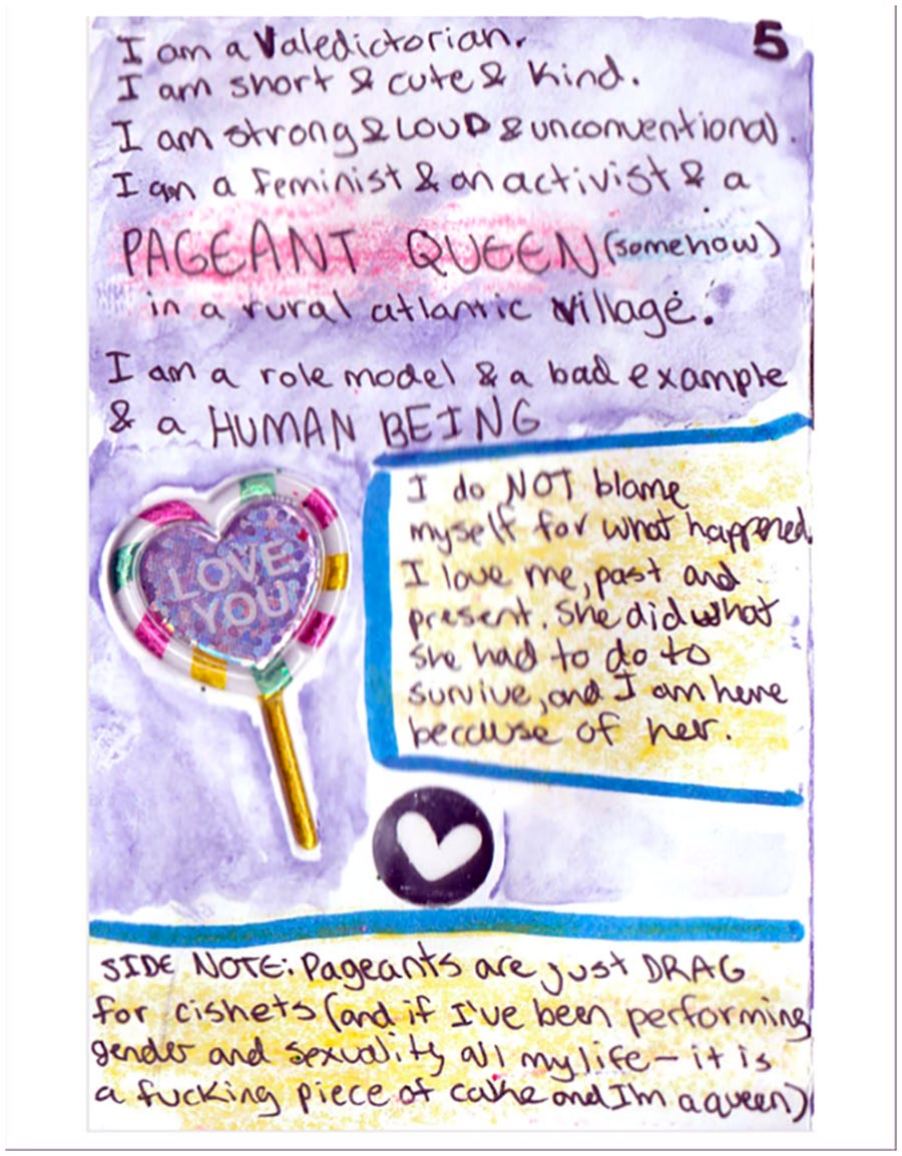
From Sarah’s *Of Duality and Contradictions* *Source*: Sarah, 2020, Fredericton: Pride/Swell

Pride/Swell included urban and rural youth working within the project, and rural expressions of queerness emerged as a central space for solidarity-building and future-making. Amelia and Casey work on unceded Wolastoqiyik territory within the context of Fredericton—‘urban’ New Brunswick—a place that has been described anecdotally as the queerest city outside of San Francisco, though we cannot find any statistics to back these claims up. There is an assumption that 2SLGBTQ+ Canadian youth go to urban centres, but what we learned in the study is that rural queer youth exist and thrive within rural spaces, too. We also know this from personal experience. Amelia—for example—grew up in rural Nova Scotia, and Casey spent much of her childhood in a small town in the Northwest Territories—though Katie grew up in Vancouver, British Columbia. We offer a page from Sarah’s zine, *Of Duality and Contradictions* (2020),^9^ which explores sexuality, gender, rurality and performance (see Figure 4). Sarah noted in a description they provided to accompany their zine, ‘I created this about my experiences as a queer youth with ADHD masking and performing gender and femininity, re-learning about myself after experiencing trauma, and being idolized in a rural community’.

**Figure 5:**
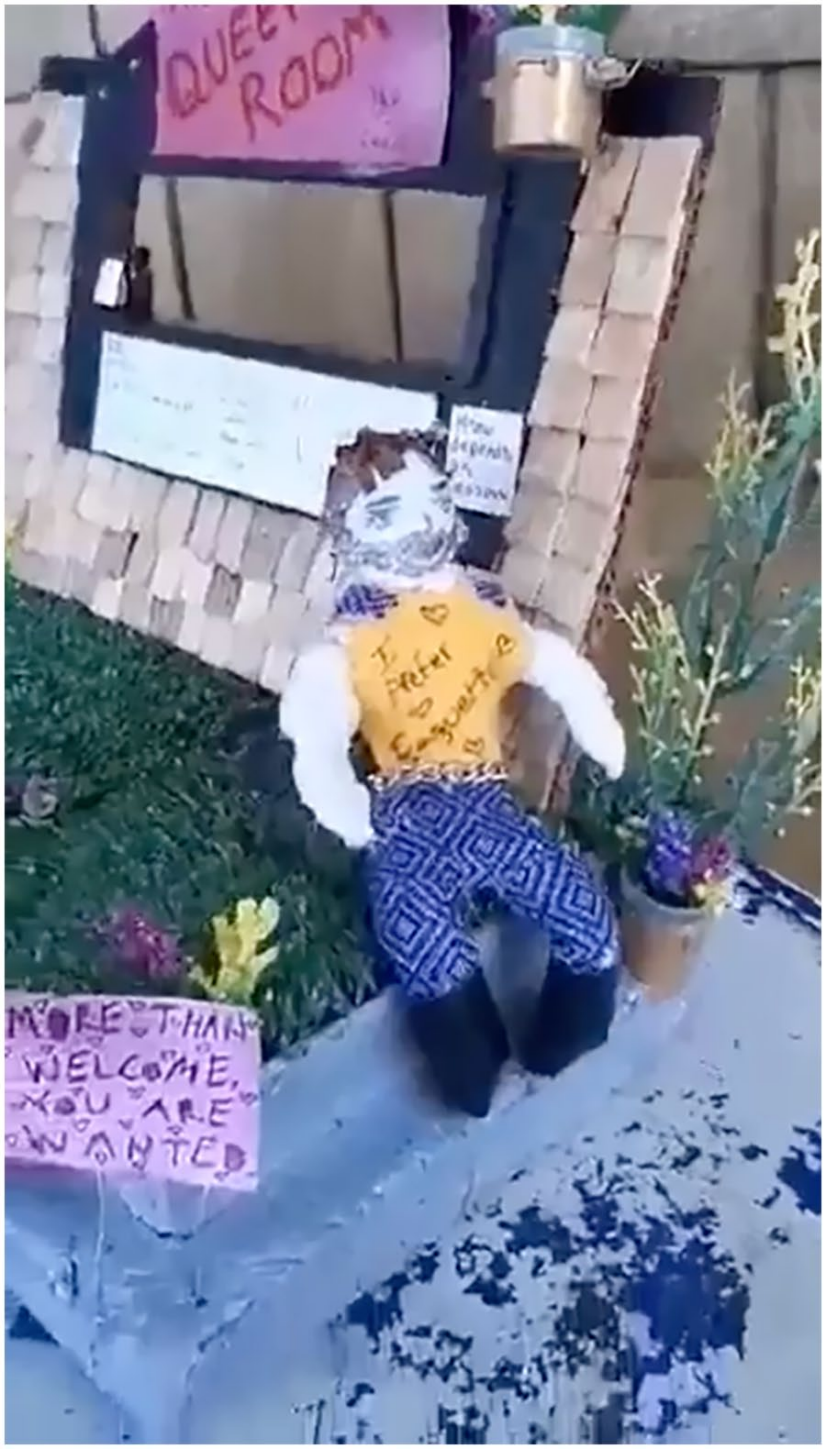
*The Queer Room* by Bliss *Source*: Bliss, 2020, Fredericton: Pride/Swell

The notion of being simultaneously queer and rural was also explored in Bliss’ diorama describing his experiences creating queer spaces within his community (see Figure 5). Bliss explained in a description that they provided with their image:

I co-founded a LGBT youth hangout place called the Queer Room about three years ago. We tried our best to make it work but it’s very hard to sustain places like that in a rural area. So, the Queer Room I’m imagining here is an elevated version. It’s a cafe for all ages by day and has a roaring nightlife by dusk. It’s absolutely necessary that this queer space be environmentally conscious. I’ve struggled with the idea of merging my environmentalism and my queer activism together. Now I realise I must. Queer people are so much more than just partying and glitter … That being said, we’re gonna party, but we’re using biodegradable glitter and decomposable cups! (Bliss, Pride/Swell programme participant)

Through Bliss’ diorama, we see the ways in which queer rural youth claim these spaces as queer.

We note that solidarities were formed through both the zines and the dioramas. Participants highlighted that they exist as joyful 2SLGBTQ+ people living within rural areas, even thriving within rural areas, and these pieces served a pedagogical function. They taught us—urban located adults and youth—about the nuanced realities of being queer and rural within Atlantic Canada.

### Networks of solidarity between older and younger participants

**Figure 6:**
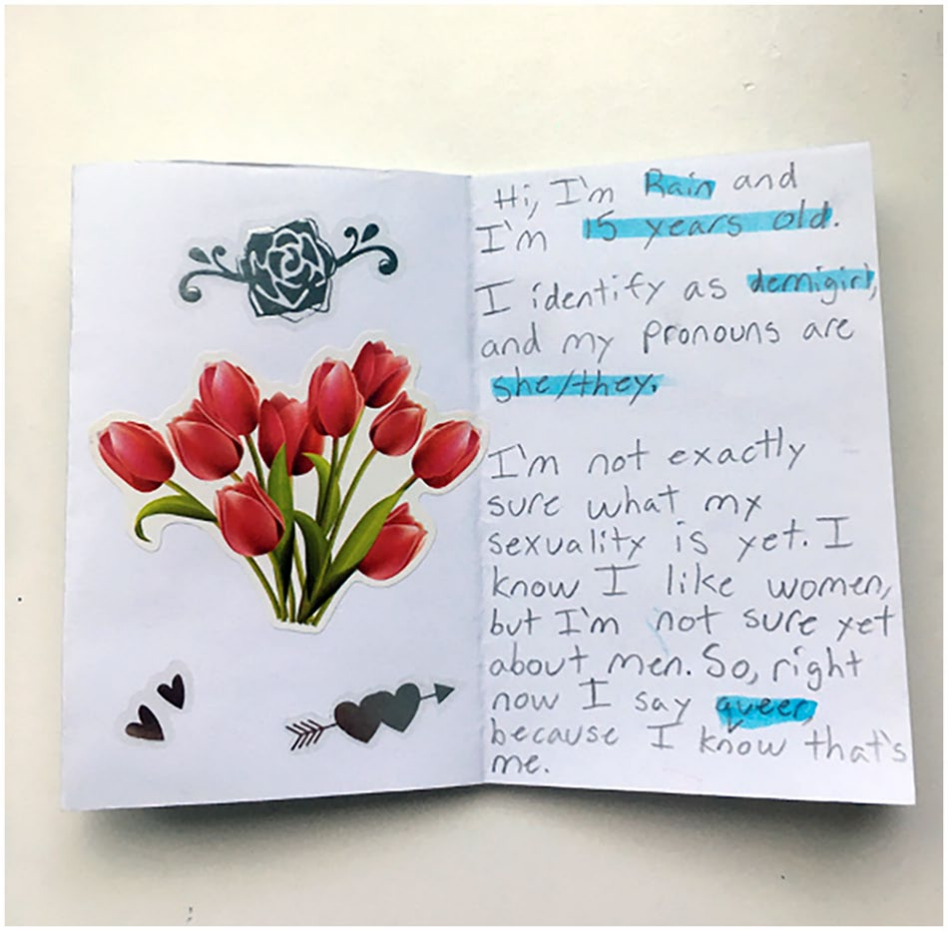
A page from Rain’s zine *Source*: Rain, 2020, Fredericton: Pride/Swell

The Pride/Swell participants ranged between the ages of 15 and 25 years old—and many were at different stages of knowing themselves. One of our younger participants, Rain, who was 15 when they made their zine, took up the notion of still figuring things out in their zine.^10^ In their zine, Rain writes, ‘I’m not exactly sure what my sexuality is yet. I know I like women, but I’m not sure yet about men. So right now I say queer because I know that’s me’. Rain’s zine (see Figure 6) explored the practice of being unsure of their attractions, and shared their feelings about comfort in claiming the term queer for themselves.

**Figure 7:**
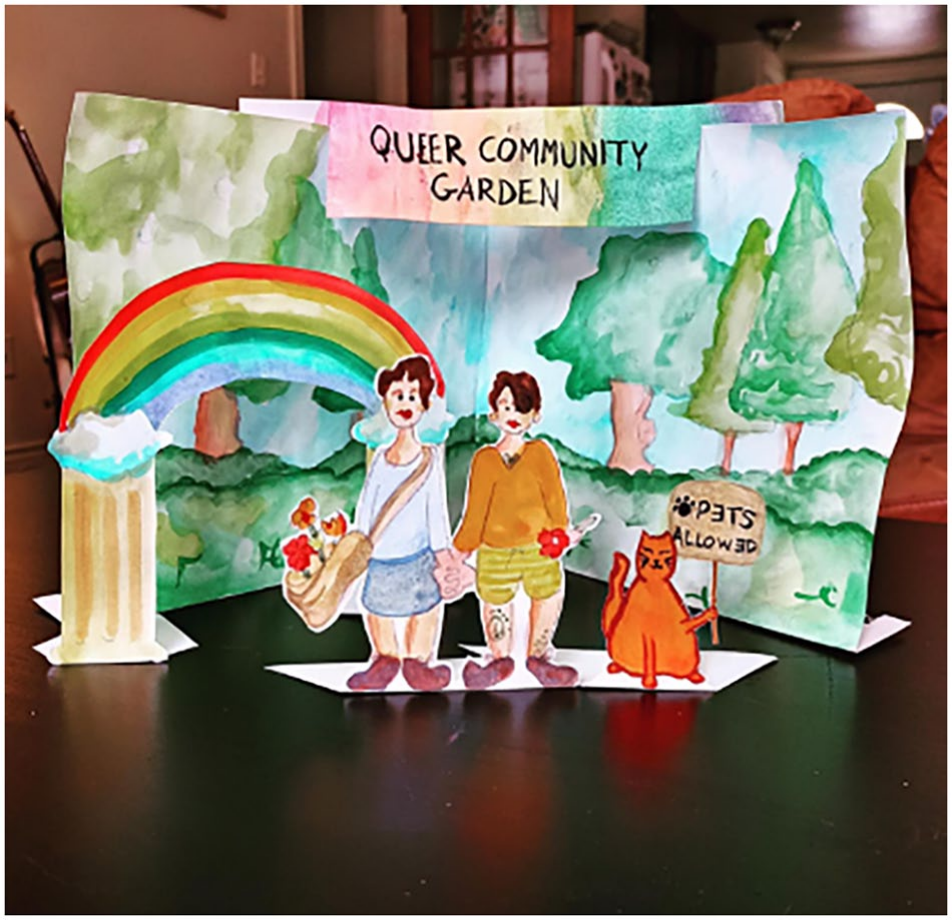
*Queer Community Garden* by Alyssa and Erin *Source*: Alyssa and Erin, 2020, Fredericton: Pride/Swell

Alyssa and Erin are a queer couple in their 20s from a rural area in Atlantic Canada who entered Pride/Swell together and co-created all their art pieces. Their imagined queer environmental future offers a watercolour community garden, with two figures holding hands (see Figure 7). Beside the couple, we see an image of a cat holding a protest sign that reads ‘pets allowed’. In sharing their adult queer life with younger participants, we see solidarities forged between those who imagine a queer adult future and those who are living it. In a Zoom meet-up where we discussed the dioramas, Bliss—a younger participant—shared, ‘I just love the way that queer adult life looks, like, how they are growing their own spaces together, and like, it is also in a small town. Like queer adult life can happen in small towns in Atlantic Canada. Like mine’.^11^ Through these burgeoning solidarities, we demonstrate different versions of adult queer life—from within the younger and older youth participants and also within the research team.

### Networks of solidarity between the research team and youth

**Figure 8:**
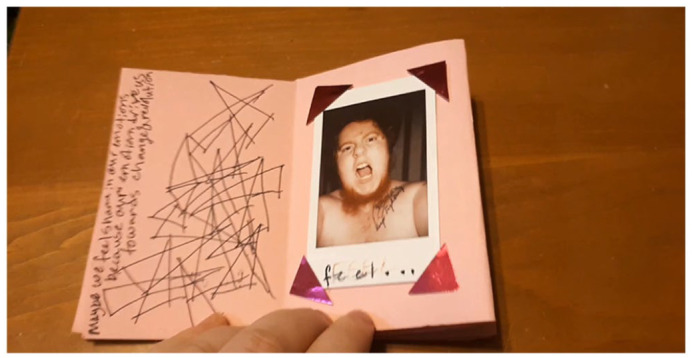
Max’s zine, *Maybe* *Source*: Max, 2020, Fredericton: Pride/Swell

Within our project, we also saw solidarities forged between research team members and participants through the sharing and co-producing of art pieces. Participants shared their zines in response to the zine that Casey created, highlighting both how to make a zine as well as her thoughts on intersectionality, queerness, identity and being closeted within her family (see also Burkholder, Hamill and Thorpe, 2021).^12^ Within their response to the zine prompt (see Figure 8), Max noted:

I made this zine while I was really overwhelmed. I was feeling a lot of things and started thinking about intersectionality. Specifically, how living at the intersections of multiple forms of oppression can sometimes lead people to have a lot of feelings that broader society would deem inappropriate. I find I am often called upon to ‘just get over it’ by people who don’t understand my struggles. To those people I have a new challenge: how about you learn to give a sh!t about other people? (Max, Pride/Swell programme participant)

**Figure 9:**
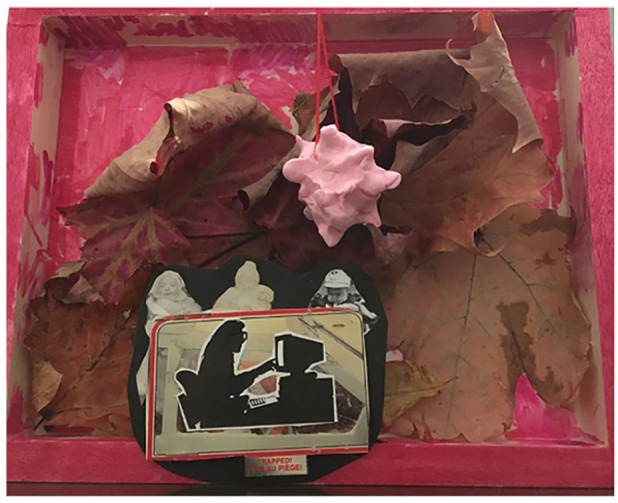
*Trapped* by Casey *Source*: Casey Burkholder, 2020, Fredericton: Pride/Swell

We saw solidarities forged between participants and the research team through co-production. Casey consistently made each art piece and shared her thoughts on each of the prompts (see also Burkholder, Hamill and Thorpe, 2021). Casey’s diorama took up issues of isolation and queer environmental futures (see Figure 9). As she exhibited her diorama, as she exhibited the piece on Instagram, Casey wrote:

I know that we are meant to imagine a future that is in the distance, but I have been having some difficulty imagining a future beyond a few weeks ahead of where I am right now. I want to take Sabine Lebel’s ideas about queering environmental futures—particularly where she asks us to queer notions of dystopia/utopia and question also notions of futurity when so much is unknown. Also, at the same time, even though I am feeling trapped in the present (and not so distant futures), and the leaves are falling and crumbling and the changing seasons bring new unknowns, I am also drawn to continue to make, to collaborate, to connect, from a distance, and so my challenge to myself is also to queer the notion of feeling trapped in this place and time and to remember to keep thinking and making and connecting and collaborating with an eye to the uncertain futures that are on their way.^13^

Through this making, and a thinking through of queer environmental futurities, as well as by sharing her work with participants in digital spaces, Casey sought to forge solidarities by highlighting her own uncertainties about futurities.

### Networks of solidarity in virtual and embodied spaces

As a research team, we also forged solidarities through connections between ourselves, our identities and our experiences, and those of participants. In September 2020, for example, at an early Zoom meet-up, Casey talked about her experiences as bisexual and closeted with her parents:

I’m Casey, as you may know, you probably know. My pronouns are also she and her, and I sent you a zine this week talking about being closeted to my parents, which is hilarious now that I’m going to have to come out to my parents before I share that online, which is really interesting to think about being old and privileged and a professor, yet still being afraid of my parents’ opinion. I’m so thankful for this project to make me think about being open and honest with the people in my life for the first time. That’s kinda nice. And I wouldn’t have done it without the zine project, so, how about that.^14^

This purposeful positioning and transparency built deep connections between the research team and participants—and also highlighted the ways in which knowledge produced within the project was never just top down, from the research team to participants.

Our Zoom meet-ups provided digital space to build solidarity across distance, engaging feminist ethics of care and community through sharing our experiences at every step of the project. The two following excerpts, from our zine and diorama meet-ups, provide insight into the active process of building solidarity and participatory analysis between the participants, the research team and guest artists. While the production of art and the curation of participatory archives were primary goals in our project, we seek to include these examples of how solidarities were developed between Pride/Swell participants, the research team and guest artists in unexpected places (like on Zoom). We begin with an excerpt from our Zoom-based zine meet-up, which has been edited for readability:

Casey: My zine was all about not … having my parents knowing my sexuality. And so, on Facebook, I recut the video of my Zine so the page that talked about being closeted was missing but on Instagram, YouTube, our website, I kept it in, because that’s like the whole point of the piece. But, I thought it was really interesting that even me, I was like, ooh I don’t want to hurt anyone’s feelings in, in sharing this in this particular space. And I know a couple of other folks who aren’t on the call now, but who sent me messages in an email saying, yeah share these pages, but not this one. […] Or share the zine here and not here. And I think it’s really important to be sort of like intentional about that kind of sharing.

Katie: Because it was the risks that also make the, it beautiful, right? I mean maybe what I read was a risk, wasn’t a risk and it was something else for somebody that was a risk that I didn’t even take up as a risk too, right? […] I don’t want to assume my risks are your risks. My trauma is not your trauma, right? [laughs] So, anyways sorry.

Fran: I think that’s the kind of one thing that I really appreciate about zines is this, this space to be vulnerable and be raw as well in your emotion as you are in like the creation of the zine as well, ’cause I mean, zines are hugely popular in activist circles for that raw, you know, easy to distribute, the way that they exist, and I think it lends itself to raw emotions as well cause, you know, it’s just so archaic in the best way possible I guess, for lack of a better, like way of describing that [laughs].

Casey: Yeah. Bliss, you got a lot of comments on your zine. Like, a ton! How did that, how did that feel? […]

Bliss: Yeah, they were all good comments and all that. […] They were all my friends that had seen it and stuff. That was pretty nice ’cause, with quarantine and all that, you don’t really communicate that much. So it was fun to see all the support coming in. Yeah, it was a great reaction that I got from people. It was really nice because it was pretty personal, so it was very affirming.^15^

In this excerpt, we see how transparency, humour and connection served to forge solidarities between members of the research team and participants.

When we met on Zoom in November 2020 to discuss diorama production, we found that solidarities were forged within the virtual space between the research team, participants and our featured guest artist, Dr Sabine LeBel, who described how her art and activism thinks through queer community, climate change and uncertain futures (see also LeBel 2022). We offer an excerpt from that Zoom call to highlight these solidarities:

Casey: I’m obviously deeply impacted by your work, Sabine, and I’m really excited about the notion of queer environmental futures as being something beyond utopia and dystopia. And I think that’s the thing that I thought would be really interesting for us to investigate as a collective of people living and working in Atlantic Canada …

Sabine: […] One of the things I think is really cool about dioramas for this prompt is, there’s a world-making element to it, which is something that I’ve been thinking a lot about with this project. And if you’re ever looking at science fiction and people who are doing writerly science fiction, there’s so much stuff around world-making. And so there’s something cool about the prompt, and, and I never would have thought to make a diorama, so there’s something really cool about how that connects.

Casey: One of the things I wanted to do was give participants natural materials from my yard and … I made mine when the leaves were fresh, but over time the leaves have crumpled and decayed. And so the piece to me is way more interesting and textural now than it was when I made it when it was much flatter for me. In my diorama, I wasn’t thinking too futuristic, I was thinking: future spring, future now, what does it mean in COVID to find sort of an environmental balance between utopia and dystopia, which is sort of, I feel like what many of us are living right now is this strange in-between and neither word feels exactly right, it’s … I don’t know. I didn’t expect so much anxiety and also simultaneously boredom during this time. It’s a very destabilising feeling and something that I hadn’t imagined in engaging in science fiction worlds for example, as a young person. So, I wanted to sort of capture this frenetic anxiety plus boredom. I sculpted a model of COVID out of tiny bits of putty that I gave everybody. And that’s me at my computer. And it says ‘trapped’. [laughs] So there we go. [laughs] That was my interpretation of the prompt. That’s where I’m living and working today. And also, thinking in the not so far ahead future. Anyone else? What were you thinking and, and feeling?

Indra: I haven’t finished mine. I’m maybe a quarter done. I ended up, I loved the putty that you gave us, and I ended up like wanting more of that texture, like squishy. I got some modelling clay that I, I think maybe my diorama rather than being upright like yours will be flat. But, is that still a diorama if it’s just like coming up this way?

Casey and Sabine: Yes!!^16^

In this excerpt, we see solidarities forming amongst the research team, the guest artist and the participants, who offer their interpretations of the prompt, thoughts on making with natural materials, and thinking through our own relationships to home during COVID-19. None of these conversations and interactions would have happened without the pandemic, the materials being sent to participants’ homes or the opportunities provided by the virtual realm.

## New opportunities for solidarities

As we reflect on Pride/Swell, we think through the ways that we built solidarities across spaces, across age ranges, through the digital and across research team/participant power structures. What did 2SLGBTQ+ solidarity and feminist futures-making look like in our project? It included ongoing and enthusiastic consent, and developing strategies to enact solidarity with personal identities in private (home) and public (digital) spaces. These are contexts that have the potential to contribute to shame or marginalisation *and* provide safety to explore and flourish. Fostering solidarity with 2SLGBTQ+ participants meant meeting youth where they were at, and creating spaces and avenues for solidarity to emerge and futures to be imagined and forged. Within our project, networks of solidarity were engaged and negotiated through the mail and online through explicitly DIY, queer and feminist methodological practices; thus, we see participatory visual inquiry through the mail as a kind of queer and feminist methodological future.

We see now that the pandemic afforded us these new ways of connecting, through the mail and online, in ways that we would not have done if we could be together. Collaborating and creating at a distance allowed us to work with more youth and in more rural and remote settings. We also saw that time and distance created disconnection in the project: though fifty-five participants began actively creating with us in August 2020, by the end of the project in July 2021 only six participants were continuing to produce and share their artworks. However, we do not see this disconnection as a failure, and that participants continued to receive packages in the mail and make them or not and display them or not is enough for us. We were able to redistribute resources from an academic grant and reach geographically isolated participants with art materials and prompts and build connections across spaces at a time when our physical movement was limited and so many of us were confined within our home spaces—practical modes of manifesting solidarity. We see Pride/Swell as an innovative way to think through solidarity-building through the mail and online, and we seek to continue engaging in this way, even as our communities shift the ways in which they approach (and ignore) the ongoing COVID-19 pandemic.

A new iteration of our project, Pride/Swell+ (2022–2023), has been working with 2SLGBTQ+ folks (ages 14 to 50+) to create art, archives and opportunities for solidarity-building via the mail and in person. We have envisioned this emerging project as a queer feminist methodological exploration: an explicitly intergenerational collaboration between queer youth and older adults in Atlantic Canada through postal mail and online. We have pivoted once more to adapt our processes to working in intergenerational spaces. We want to continue to build on our project’s successes and think through ways to build queer feminist solidarity in ways that are more antiracist, more accessible, more thoughtful, more care-focused and more grounded in our community’s needs and strengths through explicitly queer and feminist practices.

## Notes

1 2SLGBTQ+ refers to the identities Two Spirit, lesbian, gay, bisexual, transgender and/or queer, and the plus refers to the identities that are not captured in the existing acronym (e.g., intersex, asexual, etc.).

2 Participants shared their positionalities in a zine-making project where they explored their intersectional identities beyond their gender and sexuality. Many participants disclosed mental illness, physical and cognitive disabilities, and rural identities and class identities, including economic marginalisation. A more thorough explanation of these identities occurs in the findings section, below.

3 Drawing on the activism and scholarship of Eve Ewing (2020), we explicitly capitalise ‘White’, as the ‘seeming invisibility [of Whiteness] permits White people to move through the World without ever considering the fact of their Whiteness … White people get to be only normal, neutral, or without any race at all’. Foregrounding Whiteness as a category is essential in our claims of solidarity and future-making with 2SLGBT+ youth, and as we move forward as an activist community that strives to be inclusive and representational of the Atlantic context.

4 In 2022, we expanded Pride/Swell and built up our participants research team to include more Indigiqueer and trans participants; however, this new iteration is still dominated by White queer and trans participants.

5 See Facebook post, Sabine LeBel, ‘Pandemic art project’, 1 April 1 2020, https://www.facebook.com/sabine.lebel [last accessed 7 July 2023].

6 See Pride/Swell+, https://prideswell.org, https://www.facebook.com/pride.swell.art, https://twitter.com/SwellPride and https://www.instagram.com/pride.swell/ [last accessed 29 August 2023].

7 When we had a Zoom meet-up about printmaking, we hosted a speaker from Radstorm, a radical queer printmaking studio in Halifax, Nova Scotia. When we created masks, Dr Rebecca Salazar Leon, a Fredericton-based artist and activist and the co-founder of BIPOC Pride in New Brunswick, spoke about the radical possibilities of sewing.

8 Facebook post, Sabine LeBel, ‘Pandemic art project’, 1 April 1 2020, https://www.facebook.com/sabine.lebel [last accessed 7 July 2023].

9 See the full version of Sarah’s *Of Duality and Contradictions* (2020) zine at https://www.prideswell.org/zines?pgid=kfpbiilt-8d02f306-d8f2-4e0a-a3b6-badcc070ac6b [last accessed 29 August 2023].

10 See the full version of Rain’s (2020) zine at https://www.prideswell.org/zines?pgid=kfpbiilt-428ac0fb-09b1-4623-9142-09693c68f413 [last accessed 29 August 2023].

11 Pride/Swell, Zoom meeting, 24 September 2020.

12 Happily, Casey has come out to her parents since the project began (at age 37), and it is so interesting that she was working with Pride/Swell and queer youth who were navigating the coming-out space at the same time as she was herself, but much older.

13 See Instagram post, Casey Burkholder [as Pride/Swell], *Trapped*, 9 December 2020, https://www.instagram.com/p/CIk7WVLpaF-/ [last accessed 10 September 2023].

14 Pride/Swell, Zoom meeting, 24 September 2020; see also Burkholder, Hamill and Thorpe, 2021.

15 Pride/Swell, Zoom meeting, 24 September 2020.

16 Pride/Swell, Zoom meeting, 23 October 2020.
